# Determining the effects of elevated partial pressure of oxygen on hypercapnia-induced cerebrovascular reactivity

**DOI:** 10.1177/0271678X231197000

**Published:** 2023-08-26

**Authors:** Ece Su Sayin, James Duffin, Julien Poublanc, Lashmikumar Venkatraghavan, David John Mikulis, Joseph Arnold Fisher, Olivia Sobczyk

**Affiliations:** 1 Department of Physiology, University of Toronto, Toronto, Ontario, Canada; 2 Department of Anaesthesia and Pain Management, University Health Network, University of Toronto, Toronto, ON, Canada; 3 Joint Department of Medical Imaging and the Functional Neuroimaging Lab, University Health Network, Toronto, ON, Canada

**Keywords:** Cerebrovascular reactivity, MRI, hypercapnia, repeatability, hyperoxia

## Abstract

Evaluation of cerebrovascular reactivity (CVR) to hypo- and hypercapnia is a valuable test for the assessment of vasodilatory reserve. While hypercapnia-induced CVR testing is usually performed at normoxia, mild hyperoxia may increase tolerability of hypercapnia by reducing the ventilatory distress. However, the effects of mild hyperoxia on CVR was unknown. We therefore recruited 21 patients with a range of steno-occlusive diseases and 12 healthy participants who underwent a standardized 13-minute step plus ramp CVR test with a carbon dioxide gas challenge at the subject’s resting end-tidal partial pressure of oxygen or at mild hyperoxia (PetO_2_ = 150 mmHg) depending on to which group they were assigned. In 11 patients, the second CVR test was at normoxia to examine test-retest differences. CVR was defined as % Δ Signal/ΔPetCO_2_. We found that there was no significant difference between CVR test results conducted at normoxia and at mild hyperoxia for participants in Groups 1 and 2 for the step and ramp portion. We also found no difference between test and retest CVR at normoxia for patients with cerebrovascular pathology (Group 3) for step and ramp portion. We concluded normoxic CVR is repeatable, and that mild hyperoxia does not affect CVR.

## Introduction

The changes in cerebrovascular reactivity (CVR) to both hypocapnia and hypercapnia are a reliable indicator of the extent of hemodynamic disruption engendered by underlying cerebrovascular disease such as cerebral steno-occlusive disease (SOD).^1^ In patients who develop effective compensatory changes such as neovascular collateral blood flow, cerebral flow patterns may normalize, particularly at rest.^2^ Abnormalities in flow distribution may nevertheless be elicited by stimulating an increased flow demand beyond what existing compensatory vasculature can supply.

The vascular response to hypercapnia causing vasodilation and hypocapnia causing vasoconstriction have been long known.^3,4^ Cerebral vascular response with visually evoked neural activation have been measured with H_2_^15^O and positron emission tomography (PET) where regional differences ^5^ and age-related changes ^6^ were examined. Furthermore, changes in cerebral blood flow (CBF) (and cerebral blood volume (CBV)) during hypocapnia and hypercapnia were measured with H_2_^15^O, ^11^CO and PET.^7,8^ Hemodynamic behaviors have also been previously examined with magnetic resonance imaging (MRI).^9^ CVR reflects the ability of the cerebral vasculature to respond to a vasodilatory stimulus. CVR had been defined as %ΔCBF per ΔmmHg CO_2_.^10^ However, CVR is usually measured as the change in blood oxygen-level dependent (BOLD) MRI signal (Δ S) as a surrogate for the change in regional CBF to a vasodilatory stimulus in response to an increase in the end-tidal partial pressure of CO_2_ (PetCO_2_), i.e., CVR = %Δ S/Δ PetCO_2_. We have previously shown that reductions of CBF induced by increases in PCO_2_, are can identify hemodynamically significant stenosis in a major brain blood vessel indicating an enhanced risk of stroke,^11,12^ dementia,^13–15^ sickle cell disease ^16–18^ and traumatic brain injury.^19,20^ Using standardized provocative stimuli and MR measures, increases the sensitivity of the test to underlying pathology,^21^ and to changes in CVR between tests.^22,23^ Our hypercapnic breathing protocol consisted of a standardized step and ramp increase in PetCO_2_ while maintaining isoxia. The step portion provides a measure of both the magnitude and the speed of response to a 10 mmHg increase in PetCO_2_ above resting, with the speed characterized by the time constant of a fitted first-order exponential.^24^ The ramp portion of the protocol provides BOLD responses from hypocapnia to hypercapnia to both classify the pattern of response over this range of PetCO_2,_ and the magnitude of the response when the stimulus is applied sufficiently slowly to allow the response to fully evolve.^25^ Both analyses are important for assessing the efficacy of collateral blood flow in patients at risk.^1,26,27^

At our institution, a standardized hypercapnic protocol establishes a baseline BOLD signal distribution at the individual’s resting PetO_2_ and PetCO_2_ and then applies standardized isoxic, pattern of hypercapnic changes. These protocols are usually well tolerated.^28^ However, in some individuals with more sensitive respiratory chemoreflexes, the hypercapnia may result in ventilatory discomfort. Carotid body stimulation may enhance distress through its link to sympathetic drive.^29^ Hypercapnia may lead to an increase in blood pressure during the CVR test and thereby alter CBF ^30^ and hence CVR. We considered whether a mild elevation of PetO_2_ may reduce the sensitivity of the carotid body-mediated respiratory chemoreflex to hypercapnia ^31^ and thereby assuage the ventilatory distress and hypertension. A mild hyperoxic level of 150 mmHg was expected to be sufficient to silence peripheral respiratory chemoreceptors in the carotid body which by reducing the drive to breathing should reduce dyspnoea and make the increase in CO_2_ more tolerable.

We previously examined the test-retest differences between subjects and across different scanner platforms in healthy controls and found no difference in CVR.^23^ However, the repeatability of the CVR protocol in patients with SOD has not been examined. In addition, the effect of increased inspired PetO_2_ on CVR in healthy subjects and patients with cerebrovascular pathology has not been documented. Thus, the aim of this study was to: (i) Examine the difference in CVR under normoxia and mild hyperoxia in both healthy subjects and patients with SOD; (ii) Examine the test-retest difference of CVR at normoxia in patients with steno-occlusive cerebrovascular disease.

## Methods

### Ethical approval

This study conformed to the standards set by the latest revision of the Declaration of Helsinki, except for registration in a database, and was approved by the Research Ethics Board of the University Health Network (UHN) and Health Canada. The reference number for this study is CAPCR 13-6536. All participants provided written and informed consent to participate in the study and their data was anonymized according to UHN protocols. The 12 healthy participants (3 Female), (Group 1), were referred by word of mouth. The participants were between the ages of 22 and 82 (mean (SD) = 39 ± 18 years, median = 32). The healthy participants were non-smokers, not on any medication and had no known history of neurological or cardiovascular diseases. The 21 patients with known cerebral vascular disease were recruited to the study from the outpatient Neurosurgery clinic, who are being followed with CVR testing for the natural history and progress of their steno-occlusive vascular disease for research purposes. The patients were further grouped by random assignment into two groups. Group 2 consisted of 11 patients (6 female) who underwent CVR at normoxia and again at hyperoxia. Group 3, consisted of 10 patients (6 Female) who underwent both CVR studies at normoxia. The participant demographics are displayed in Table 1 and patient characteristics are available in Supplementary Table 1.

**Table 1.** Participant demographics.

	Age range (y)	n	Sex (M:F)
Group 1: Healthy ParticipantsCVR 1) resting PETO_2_ & 2) PETO_2_ 150 mmHg	18–35	7	4:3
	36–60	4	4:0
	61–85	1	1:0
	Total	12	9:3
Group 2: Patient GroupCVR 1) resting PETO_2_ & 2) PETO_2_ 150 mmHg	18–35	3	1:2
	36–60	4	0:4
	61–85	3	1:2
	Total	10	2:8
Group 3: Patient GroupCVR 1) & 2) at resting PETO_2_	18–35	3	1:2
	36–60	7	4:3
	61–85	1	0:1
	Total	11	5:6

### Experimental protocol

For the CVR test, a facemask was fitted to the participant’s face using skin adhesive (Tegaderm, 3 M, Saint Paul, MN, U.S.A.) and inspired gas provided via an automated programable gas blender (RespirAct™, Thornhill Medical, Toronto, Canada) which controlled PetO_2_ and PetCO_2_ independently of each other and independent of subject ventilatory pattern via a prospective end tidal gas targeting algorithm.^32^ All subjects received the same hypercapnic paradigm using their individual resting PetCO_2_ as the baseline for each successive CVR.

**Figure 1.**
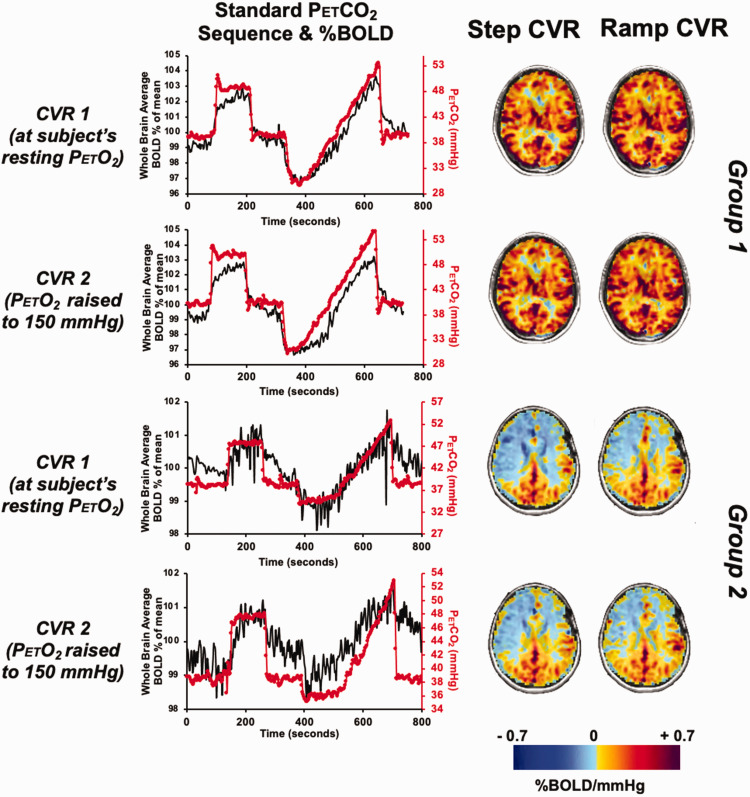
The standardized BOLD and PetCO_2_ graph and their corresponding CVR maps for Groups 1 and 2. Comparisons of CVR maps in two representative participants, one from group 1 and one from group 2. Graphs show the PetCO_2_ sequence protocol (red) and whole brain average BOLD (% of mean, black). Scans show same slice CVR for step portion on the left, ramp portion on the right; normoxia upper row and hyperoxia corresponding lower row.

The PetCO_2_ sequence in all individuals consisted of manipulating the PetCO_2_ over a 13-minute and 20 second standardized step and ramp pattern (Figure 1). This stimulus consisted of 1) clamping PetCO_2_ at the individual’s respective resting PetCO_2_ for 2 minutes, 2) a step increase of 10 mmHg for 2 minutes, 3) a return to the subject’s baseline for another 2 minutes, 4) a reduction of PetCO_2_ by 10 mmHg from the resting for 1 minute, 5) followed by a steady rise (ramp) to 15 mmHg above resting over a 4.2-minute period, and 6) a return to baseline for 2 minutes. A brachial cuff was placed on each individual and blood pressure was measured manually every minute during both CVR tests using the non-invasive blood pressure portion of an MRI compatible patient monitoring system (Invivo Expression System; Invivo Corporation, Orlando, FL, USA). Blood pressure measurements were sampled every 1 minute in all participants during both CVR acquisitions.

All participants underwent 2 CVR tests at the same MRI session with 20 minutes between CVR tests. For Groups 1 and 2, their respective resting PetO_2_ was targeted at normoxia for the first CVR and then a PetO_2_ of 150 mmHg was targeted for the second CVR. The PetO_2_ target of 150 mmHg was maintained for 2 minutes prior to the start of the CVR test. Group 3 followed the same normoxic protocol during each CVR (test-retest).

### Data acquisition

The scans were acquired on a 3-Tesla scanner (HDx Signa platform, GE healthcare, Milwaukee, WI, USA) with an 8-channel head coil. The same fast spoiled gradient echo (FSPGR) T1-weigthed scan and two BOLD sequences with identical scanning parameters that were each 13 minutes and 20 seconds long, were acquired for all participants. The FSPGR scan was a high-resolution T1-weighted scan with a 3D spoiled gradient echo sequence. The following parameters were used: TI = 450 ms, TR 7.88 ms, TE = 3 ms, flip angle = 12°, voxel size = 0.859 × 0.859 × 1 mm, matrix size = 256 × 256, 146 slices, field of view =24 × 24 cm, no interslice gap. Next, the two BOLD sequences consisted of a T2*-weighted echoplanar imaging gradient during the PetCO_2_ and PetO_2_ manipulation. The following sequence parameters were used: TR = 2400 ms, TE = 30 ms, flip angle = 85°, 41 slices, voxel size = 3.5 mm^3^ and matrix size = 64 × 64.

### Data analysis

The acquired MR images were imported into Analysis of Functional NeuroImages (AFNI) software (National Institutes of Health, Bethesda, Maryland).^33^ The BOLD signals from the two CVR tests were volume registered, slice-time corrected and co-registered to the anatomical images. The high-resolution T1-weighted (FSPGR) images were segmented into gray matter (GM) and white matter (WM) using SPM8 (Wellcome Department of Imaging Neuroscience, Institute of Neurology, University College, London, UK). The CVR was analyzed in an identical manner for all 3 groups. First, to account for the delay in gas sample arrival at the sensor, the PetCO_2_ values were first time-shifted to maximum correlation with the whole brain average BOLD signal. The signal was broken into two parts: the step and ramp portion of the protocol. For each part of the protocol, on a voxel-by-voxel basis, a linear fit of the BOLD signal data series to the PetCO_2_ data series was performed with a linear trend regressor to calculate step and ramp CVR values. CVR is defined as the ratio of the change in BOLD MRI signal to an increase in blood PCO_2_: % Δ Signal/Δ PCO_2_, expressed in %/mmHg.^24,34^ The individual specific GM and WM masks were used to calculate average GM and WM values for the CVR measures. The calculated CVR values were overlayed on the segmented T1-weigthed images and color-coded to a color scale to display CVR maps.

### Statistical analysis

Using SigmaStat (Systat Software, San Jose, California, USA), a Friedman One-Way Repeated Measure Analysis of Variance (rmANOVA) by ranks was performed when the normality test (Shapiro-Wilk) failed to determine significant difference between resting PetCO_2_ and PetO_2_ from the first CVR to the second for all groups. Threshold-free cluster enhancement (TFCE) analysis was used to determine if significant differences existed between the two CVR scans using a voxel-wise paired t-test (α = 0.05). A “randomise” permutation-based analysis was then applied to the height of the maxima of the resulting statistic image, using the ‘randomise’ permutation-based inference tool (Winkler et al., 2014) in FSL v.5.0.9 (FMRIB Library https://fsl.fmrib.ox.ac.uk/fsl/fslwiki/) that allowed for the maintenance of strong control over family-wise error. To evaluate the CVR agreement between the two tests within the same individual, Lin’s concordance correlation coefficient was calculated. A one-way rmANOVA was performed to compare the CVR in the first as compared to the second test and resting, step and ramp mean arterial pressure (MAP) between tests.

### Data availability statement

Anonymized data will be shared by request from any qualified investigator for purposes such as replicating procedures and results presented in the article provided that data transfer is in agreement with the University Health Network and Health Canada legislation on the general data protection regulation. All data supporting the results in this paper are provided as Supporting Information for Online Publication.

## Results

### Resting PETCO_2_ and PETO_2_ between two CVR scans

To determine consistency and accuracy between the two CVR tests for each participant in all groups, the resting PetCO_2_ and PetO_2_ between two CVR scans were compared (Table 2). The resting PetCO_2_ for Group 1 was not significantly different (p = 0.339) between the two tests, while resting PetO_2_ was significantly higher for the second hyperoxic CVR test (p < 0.0001). The participants in Group 2 did not have a significant difference of resting PetCO_2_ (p = 0.520) between the two tests, while resting PetO_2_ was significantly higher for the second CVR (p < 0.0001). For Group 3, the resting PetCO_2_ and PetO_2_ was not significantly different between the two CVR scans (p = 0.209, p = 0.115, respectively).

**Table 2.** The average (SD) resting PetCO_2_ and PetO_2_ in mmHg for the first and second CVRs for the three groups.

	First CVR		Second CVR	
	Resting PetCO_2_	Resting PetO_2_	Resting PetCO_2_	Resting PetO_2_
Group 1 (n = 12)	39.2 (2.8)	117.6 (10.5)	39.3 (2.7)	152.8 (2.8)
Group 2 (n = 10)	38.2 (3.7)	115.0 (7.8)	38.5 (3.6)	153.0 (3.2)
Group 3 (n = 11)	37.7 (3.6)	116.2 (7.4)	38.5 (2.5)	118.2 (2.5)

### CVR comparison in groups 1 & 2

**Figure 2.**
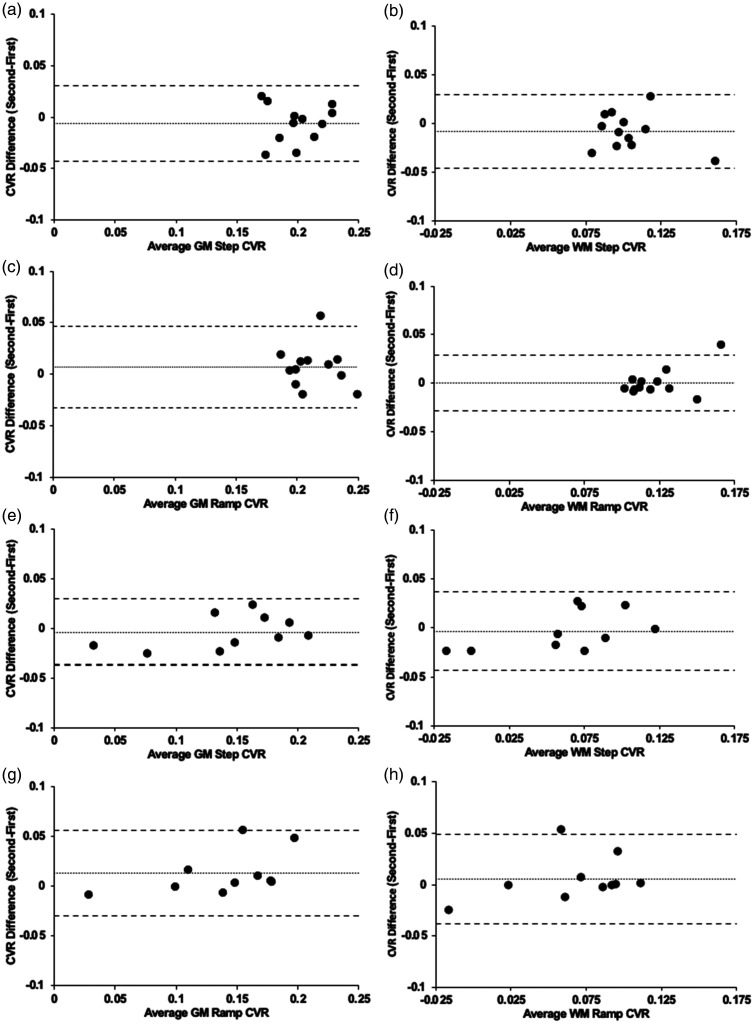
Bland-Altman plots comparing the first and second CVR differences. (a) average GM step CVR for Group 1 (n = 12) (*p = 0.295*) (b) average WM step CVR for Group 1 (p = 0.175) (c) average GM ramp CVR for Group 1 (*p = 0.279*) (d) average WM ramp CVR for Group 1 (p = 0.889) (e) average GM step CVR for Group 2 (n = 10) (*p = 0.508*) (f) average WM step CVR for Group 2 (*p = 0.662*) (g) average GM ramp CVR for Group 2 (*p = 0.889*) and (h) average WM ramp CVR for Group 2 (*p = 0.474*). Note: Solid line depicts bias, and the dashed lines show the 95% upper and lower limits of agreement as the mean difference. CVR in %BOLD/mmHg.

Figure 1 displays the standardized PetCO_2_ sequence and the respective CVR map for both the step and ramp portion of the CVR protocol in two representative participants, one from group 1 and one from group 2, respectively. A TFCE analysis paired t-test (α = 0.05) found no difference between the first and second CVR for the step and ramp CVR scans for Groups 1 and 2 (p < 0.05). The results of the Bland-Altman analysis between CVR-step and CVR-ramp for each of the participants are shown in Figure 2. The bias, scatter of points varying considerably across subjects and having a few outliers suggest there is no bias or difference in one method to the other. The calculated Lin’s correlation coefficient demonstrated moderate concordance for Group 1 and excellent concordance for Group 2 between the two CVR tests (Supplementary Table 2).

### CVR comparison in groups 3 (test re-test differences) at normoxia

**Figure 3.**
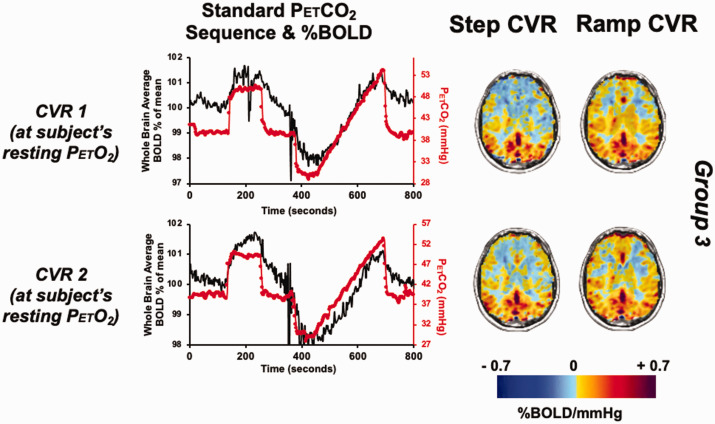
The standardized BOLD and PetCO_2_ graph and their corresponding CVR maps for Group 3. The graphs show the PetCO_2_ sequence protocol (red) and whole brain average BOLD (% of mean, black) in a representative subject from Group 3. Scans show same slice CVR for step portion on the left, ramp portion on the right; upper and lower rows are both at normoxia.

**Figure 4.**
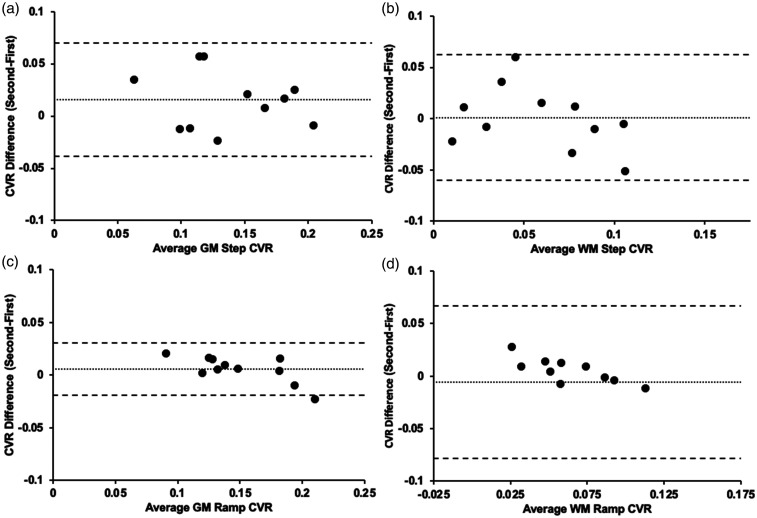
Bland-Altman plots comparing the first and second CVR differences. a) average GM step CVR for Group 3 (n = 11) (*p = 0.094*) b) average WM step CVR for Group 3 (*p = 0.991*) c) average GM ramp CVR for Group 3 (*p = 0.184*) d) average WM ramp CVR for Group 3 (*p = 0.763*). Note: Solid line depicts bias, and the dashed lines show the 95% upper and lower limits of agreement as the mean difference.

The standard PetCO_2_ sequence and the respective CVR map for both the step and ramp portion of the CVR protocol in representative participant in Group 3 is shown in Figure 3. A voxel-wise TFCE analysis paired t-test (α = 0.05) found no significant difference between two CVRs for the step and ramp CVR scans for Group 3 (p < 0.05). The results of the Bland-Altman analysis for between CVR-step and CVR-ramp comparison for each of the participants are shown in Figure 4. The bias, scatter of points varying considerably across subjects and having a few outliers suggest there is no bias or difference in one method to the other. Lin’s correlation coefficient demonstrated moderate concordance for Group 3 between the two CVR tests (Supplementary Table 2).

### Changes in blood pressure

The MAP values and their p-values are reported in Table 3.

**Table 3.** Average (SD) mean arterial pressure (MAP) (mmHg) and p-values during relevant time points of the stimulus for the first and second CVRs for each of the three groups.

	Group 1(n = 10)	Group 2(n = 12)	Group 3(n = 11)
Average MAP at baseline PetCO_2_
First CVR	84 (9.54)	90 (9.16)	90 (10.64)
Second CVR	86 (10.47)	87 (10.17)	89 (9.22)
*p*-value	*0.36*	*0.04*	*0.78*
MAP during step change in PetCO_2_
First CVR	89 (10.30)	97 (12.33)	98 (11.14)
Second CVR	93 (12.89)	92 (13.54)	92 (7.69)
*p*-value	*0.05*	*0.02*	*0.01*
Average MAP during ramp change in PetCO_2_
First CVR	87 (13.07)	94 (12.56)	95 (10.68)
Second CVR	91 (16.87)	93 (14.24)	96 (7.81)
*p*-value	*0.04*	*0.65*	*0.57*

## Discussion

The main findings of this study is that both in healthy controls and patients with SOD, there was no significant differences between a CVR test done at the subjects resting PetO_2_ and mild hyperoxia (PetO_2_ =150 mmHg). Furthermore, there was no significant difference between a repeated CVR test conducted at normoxia in patients with SOD.

As a result of this study, we have the confidence that a CVR test performed under mild hyperoxia would still be comparable to previous tests performed under normoxia. This data provides new information as to the degree of confidence available in the stability and reproducibility of CVR testing in patients with SOD tested with the same extent and pattern of vasoactive stimulus.

CVR testing with BOLD as a surrogate for CBF requires that PetO_2_ remains constant during the PetCO_2_ changes, as changes in arterial PetO_2_ may affect BOLD without commensurate changes in blood flow. As to the purpose of the study, we argue that if CVR is not affected, a PetO_2_ of 150 mmHg which is known to suppress the peripheral chemoreceptors ^35^ may be applied during testing on the chance that it may dampen some respiratory discomfort accompanying hypercapnia. Notably, we found hyperoxia does not substantially affect the CVR. These findings support the optional supplementation of the PetO_2_ during CVR for patients that may require increased inspired PetO_2_ to treat hypoxia due to pulmonary shunting associated with lung pathology such as atelectasis or COVID^36^ with some confidence that the data remain comparable to previous or subsequent studies at normoxia.

CVR differences between GM and WM are presented visually (Figures 1 and 3) and numerically from the Bland Altman plots (Figures 2 and 4) and are congruent with those previously documented.^37–39^ The ability to distinguish between the GM and WM allows for further exploration of diseases effecting vascular impairment and potential biomarkers. In particular, the ability to assess WM allows for the identification of individuals at risk of vascular dementia.^40^

Although we found that resting MAP values were not different between any of the CVR tests for all groups (Table 3). MAP at rest, during the step and ramp decreased between the first and second CVR test in the patient groups (Group 2 and 3). Average MAP during the ramp change in PetCO_2_, increased for the second CVR in Group 3. Reduced anxiety from familiarity with the protocol as well as carotid body suppression by hyperoxia may be contributing factors. However, for Group 3, the MAP at rest, during the step and ramp change in PetCO_2_, increased in the second CVR test compared to the first. Perhaps these participants were stressed due to the duration of the CVR test.

Hyperoxia had previously been shown not to have a significant effect on CBF,^41–44^ consistent with our current finding. The small differences that hyperoxia may cause in CVR would result from the expected sparing effect of the oxygen dissolved in plasma on the development of venous deoxyhemoglobin (dOHb), but the overall effect is likely too small to be measured. Another implication of the sparing effect of hyperoxia is that the PETO_2_ must remain constant during CO_2_-induced CVR.

The increase in arterial oxygen partial pressure to 150 mmHg will increase the dissolved oxygen in the plasma resulting in a trivial increase in the arterial oxygen content and no effect on the venous oxygen saturation which is the origin of the BOLD signal.^45,46^

### CVR protocol

Our standardized hypercapnic breathing protocol consists of a step and ramp increase in PetCO_2_ while maintaining isoxia. This 13-minute protocol is broken down into the step and ramp portion. We suggest not calculating CVR from the combined step and ramp protocol as there is a delay in the BOLD response during the rapid step increase in CO_2_ compared to the slower CO_2_ increase during ramp.

With a step increase in PetCO_2_ of 10 mmHg above resting, vasodilation does not occur immediately but slowly increases to its full extent as vasodilatory reserve permits. Consequently, CBF rises slowly to its steady state value. We model this CBF response measured with BOLD as a first order exponential, determining the time constant to characterize the speed of the vasodilatory response.^24^ With respect to the CVR calculation during the step stimulus, we note that the regression includes the entire rise to steady state rather than simply the difference between baseline and the steady state. Inclusion of the early rise in the BOLD signal when the CO_2_ stimulus is already at its maximum reduces the slope of the linear regression. CVR will be calculated lower than its true value.

By contrast, the ramp increase in PetCO_2_ from hypocapnia to hypercapnia is assumed to be slow enough to allow all regions of the cerebral vasculature to respond fully.^25^ Moreover, the range of PetCO_2_ during the ramp is considered to produce close to a full range of vasoconstriction to vasodilation. The entire ramp response can be analyzed in several ways^47,48^ to reflect different physiological processes. The calculation of CVR, includes the slope of the regression of the resting plus 10 mmHg PetCO_2_ portion of the ramp stimulus. This portion of the ramp is chosen to be comparable with the same change in PetCO_2_ as the step CVR.

This explains why the slow ramp increase in PetCO_2_ results in CVR that is typically greater than that of the step CVR. This difference between step and ramp CVR is an indicator of a slowed vasodilatory response. Thus, measurement of the speed of the response with the step. and the magnitude of the response with the ramp, are important for assessing the efficacy of collateral blood flow in patients at risk.^1,26,27^

### Study design

This study was designed so that every subject was their own control. Group comparisons or conclusions were avoided as it is nearly impossible to match pathology in clinical patients to create two groups that are “identical”. Even though each subject is different from the other in terms of degree and pattern of stenosis and resting arterial gas concentrations, no significant differences between the CVR tests were found. It should be noted that the study was not designed to evaluate differences in absolute CVR between the tests.

### Limitations

The effects of mild hyperoxia on comfort level of the participants was not evaluated in this study. We are less certain of any changes in ventilatory discomfort under mild hyperoxia due to the difficulty the subjects had in making such assessments as previously shown by Kahneman and Fredrickson.^49^ The order of the CVR’s performed at normoxia and mild hyperoxia for Group 2 was not randomized as our current database consists of CVRs deployed at the subjects respective resting PetO_2_.^28^ The progression of disease in most of our patient population is closely monitored and we did not want to introduce mild hyperoxia into their routine CVR scans without understanding the full effects on the magnitude of CVR. Due to the risk of the subject declining a second CVR or intolerability, the first CVR was always done at normoxia followed by a CVR done at mild hyperoxia. This order of testing is unlikely to affect the outcome of the study, but we were unable to confirm that due to clinical limitations. The test-retest reliability and effect of hyperoxia may be different in the older patients or in patients with more severe disease however the study was too small to evaluate this. In addition, the blood pressure was taken with an automated sphygmomanometer at 1-minute intervals. A continuous blood pressure monitoring device would have provided better temporal resolution.

With its non-invasiveness, high spatial and temporal resolution, and general availability, BOLD MRI methods have been shown to be favorable compared with other measures of CBF such as PET and arterial spin labeling (ASL).^50,51^ BOLD MRI contrast arises from the magnetic susceptibility of dOHb in the voxel. Changes over a short time course of a minute, or seconds, are due primarily to corresponding changes of the rate of inflow of arterial blood, which dilutes or displaces dOHb in desaturated blood in capillaries and veins.^52^ The relationship between CBF-based CVR and BOLD-based CVR is well-known to be nonlinear.^53^ Fractional BOLD signal change, plotted as a function of fractional CBF change, calculated by Hoge et al.^53^ predicts a function with a linear domain for increases in perfusion up to approximately 50%, after which it becomes nonlinear. In our study PCO_2_ increases of about 10 mmHg has been predicted to increase CBF by about 50%.^54^ Cerebral blood volume changes concurrently with flow, but the changes in volume of dOHb are very small compared to the effects of dilution by up to a 50% increase in CBF.^55^ In our study the inflowing blood was fully saturated under both normoxia and hyperoxia precluding to no differences in BOLD signal. Finally, the PCO_2_ in the normoxic and hyperoxic conditions are the same, so the small (if any) effect of PCO_2_ on CMRO_2_ and thereby on the BOLD signal would also be the same. Neuronal activation does occur during the duration of the BOLD MRI acquisitions, however we are unable to separate global CO_2_ stimulus effects from neuronal activation and have it subsumed in our measurements, including any changes in CBV or CMRO_2_.

## Conclusion

We examined the difference between deploying a CVR test at mild hyperoxia (PetO_2_ of 150 mmHg) rather than the subjects respective resting PetO_2_ in patients and healthy controls. There was no significant difference in CVR between the first and second acquisition regardless of the PetO_2_ level. In addition, CVR test-retest difference in patients with steno-occlusive disease repeatability was reliable.

## Supplemental Material

sj-pdf-1-jcb-10.1177_0271678X231197000 - Supplemental material for Determining the effects of elevated partial pressure of oxygen on hypercapnia-induced cerebrovascular reactivity Supplemental material, sj-pdf-1-jcb-10.1177_0271678X231197000 for Determining the effects of elevated partial pressure of oxygen on hypercapnia-induced cerebrovascular reactivity by Ece Su Sayin, James Duffin, Julien Poublanc, Lashmikumar Venkatraghavan, David John Mikulis, Joseph Arnold Fisher and Olivia Sobczyk in Journal of Cerebral Blood Flow & Metabolism
