# Supplementary material for: Determining the effects of elevated partial pressure of oxygen on hypercapnia-induced cerebrovascular reactivity
Source: J Cereb Blood Flow Metab. 2023 Aug 26;43(12):2085–95. doi: 10.1177/0271678X231197000 (PMC10925865; doi:10.1177/0271678X231197000)
Supplement: sj-pdf-1-jcb-10.1177_0271678X231197000 - Supplemental material for Determining the effects of elevated partial pressure of oxygen on hypercapnia-induced cerebrovascular reactivity [file sj-pdf-1-jcb-10.1177_0271678X231197000.pdf]

Supplementary Table 1. Patient demographics and interim history for participants in Groups 2 and 3.

Participant ID	Age	Sex	Interim History
**Group 2: Patient Group CVR at 1) at subject's resting PO_2 & 2) PO_2 raised to 150 mmHg**			
Participant ID	***Age***	***Sex***	***Interim History***
1	71	F	Right carotid artery dissection (type A) but adequate distal perfusion. Intermittent tingling mainly in the first digits of her left hand.
2	38	F	Diagnosed with Moyamoya Disease. Complete occlusion of right middle cerebral artery with multiple small collaterals. Underwent right EC-IC bypass. Frequent bilateral headaches.
3	81	F	Hemodynamic TIAs on the right side for a known left ICA occlusion. Underwent bypass surgery of the left middle cerebral artery to the left superficial temporal artery. Improved headaches and pain.
4	29	F	Diagnosed with bilateral, severe Moyamoya disease. Underwent left flow augmentation EC-IC bypass revascularization. Right-side continues to exhibit TIA-like symptoms.
5	35	F	Diagnosed with bilateral, severe Moyamoya disease. Underwent a left hemispheric revascularization with an EC-IC bypass. Fluctuating neurological complications.
6	75	M	Significant left distal ICA stenosis in an isolated artery. Right-sided focal numbness with or without weakness eventually caused by a lacunar infarct.
7	56	F	Left middle cerebral artery TIAs caused by underlying severe bilateral intracranial atherosclerosis.
8	50	F	A small chronic lacunar infarct is seen within the posterior aspect of the right putamen. Prior stroke and TIA (>10 years).
9	47	F	Severe stenosis of the supraclinoid left internal carotid artery with narrowing of the let anterior cerebral artery, absent anterior communicating artery and the proximal middle cerebral artery to the trifurcation.
10	34	M	Left M1 occlusion and possible Moyamoya syndrome. Recurrent right sided TIA.
**Group 3: Patient Group CVR at 1) at subject's resting PO_2 & 2) at subject's resting PO_2**			
Participant ID	***Age***	***Sex***	***Interim History***

1	44	M	Bilateral Moyamoya-like carotid stenosis and history of a right hemispheric stroke. Underwent a STA to MCA EC-IC bypass.
2	25	M	Left-sided Moyamoya secondary to neurofibromatosis type 1. Episode of pain in the back of his head associated with right hand cramping.
3	56	M	Bilateral ICA stenosis, more severe on the right side (M1 stenosis). Right hemorrhagic stroke on the right side and currently has left-sided weakness and intermittent difficulty in speaking.
4	60	F	Diagnosed with Moyamoya disease. Underwent STA to MCA bypass and has had 2 postoperative infarcts in the left hemisphere.
5	35	F	Diagnosed with bilateral Moyamoya disease and distal right internal carotid artery (occlusion is distal to the posterior communicating the distal right MCA).
6	36	F	Left MCA stenosis and recurrent left MCA infarcts.
7	44	M	Left M1 thrombus with partial occlusion. Underwent left EC-IC bypass. The occlusion of the left MCA is unchanged.
8	49	M	Known bilateral Moyamoya disease and detected ischemic lesions on MRI. Previously had a left fronto-parietal stroke
9	25	F	Known Moyamoya disease with revascularization surgery (> 10 years ago). Patent STA-MCA anastomosis. Occlusion of the internal carotid artery distal to the left posterior communicating artery.
10	81	F	Hemodynamic TIAs on the right side for a known left ICA occlusion. Underwent bypass surgery of the left middle cerebral artery to the left superficial temporal artery.
11	41	F	Known Moyamoya disease. Underwent left and right EC-IC bypass surgery

Supplementary Table 2. Calculated Lin's correlation coefficient (LCC) and the 95% confidence interval (CI) in GM and WM for the step and ramp portion of the protocol for all groups.

	Step					
	GM			***WM***		
	LCC	***95% CI***		***LCC***	***95% CI***	
		Upper	***Lower***		***Upper***	***Lower***

**Group 1 (n=10)**	0.63	0.142	0.872	0.63	0.171	0.865
**Group 2 (n=12)**	0.95	0.837	0.985	0.89	0.688	0.965
**Group 3 (n=11)**	0.77	0.389	0.926	0.65	0.169	0.881
	**Ramp**					
	GM			***WM***		
	LCC	***95% CI***		***LCC***	***95% CI***	
		Upper	***Lower***		***Upper***	***Lower***
**Group 1 (n=10)**	0.54	0.014	0.831	0.75	0.432	0.904
**Group 2 (n=12)**	0.88	0.638	0.961	0.84	0.516	0.954
**Group 3 (n=11)**	0.93	0.83	0.973	0.57	0.273	0.77
